# Liquid platelet‐rich fibrin in root surface biomodification during gingival recession treatment: Randomized, controlled, split‐mouth, clinical trial

**DOI:** 10.1002/cre2.747

**Published:** 2023-05-10

**Authors:** Wajeha Albatal, Tarek Qasem, Yasser Alsayed Tolibah

**Affiliations:** ^1^ Department of Periodontology, Faculty of Dentistry Damascus University Damascus Syria; ^2^ Department of Pediatric Dentistry, Faculty of Dentistry Damascus University Damascus Syria

**Keywords:** biomodification, free gingival graft, gingival recession, liquid platelet‐rich fibrin

## Abstract

**Background and Objective:**

Free gingival graft (FGG) has been successfully used in the treatment of gingival recessions, as it is the most predictable technique for increasing the attached gingiva. This study aimed to evaluate the effect of liquid platelet‐rich fibrin (PRF) with FGG on root surface coverage as root surface biomodification.

**Materials and Methods:**

The research sample consisted of 32 surgical sites in 16 patients, they had 2 bilateral recessions in the incisor area of the same dental arch, the sample was divided into 2 groups randomly, and liquid PRF was applied in the first group with the FGG (experimental group), and in the second group the FGG was applied alone (control group). Gingival recession depth (RD) and width of attached gingiva (WAG) were measured before starting, after 1, 3, and 6 months. The percentage of root coverage (RC) was calculated after 6 months. Healing Index (HI) was recorded after 1 week, 2 weeks, and 1 month.

**Results:**

Both groups showed a reduction in gingival RD during all follow‐up periods but the difference between both groups was not statistically significant (*p* > 0.05) at 1 and 3 months, whereas there were significant differences at 6 months (*p* = 0.001). RC was better in the liquid PRF group than in the control group, but this difference was not statistically significant (*p* > 0.05). The postoperative 7th and 14th days HI scores of the liquid PRF group were significantly better than the control group (*p* = 0.000 and *p* = 0.004, respectively), whereas there were no significant differences in HI scores between both groups at first month (*p* > 0.05).

**Conclusions:**

According to the results, the addition of liquid PRF to the root surface with FGG showed further development in terms of decreasing RD, increasing WAG, and accelerated wound‐healing.

## 1 INTRODUCTION

Gingival recession (GR) is defined as a displacement of the gingiva apically from the cementoenamel junction (CEJ), thus exposing the root surface (Lindhe et al., 2008). It is a common phenomenon in populations with high and poor standards of oral hygiene, it increases with age and it is greater in men than in women of the same age, and it is more severe at buccal than at interproximal surfaces of teeth, although of that, some patients are unaware of this condition, but it may be important in many cases due to its esthetical problems, hypersensitivity, and root caries (Kassab & Cohen, 2003). There are many primary factors responsible for GR, like low levels and long‐lasting trauma, such as inappropriate daily brushing, chronic inflammatory periodontal disease, periodontal treatment, and occlusal trauma, whereas the decreased alveolar bone crest thickness, combined with delicate gingival margin, dehiscences, and frenulum insertion are the predisposing factors (Jati et al., 2016). Several surgical techniques have been reported to treat GR such as lateral pedicle flap, free gingival graft (FGG), coronally advanced flap (CAF), semilunar coronally repositioned flap, and subepithelial connective tissue graft (SCTG), thrombocyte‐rich fibrin, and acellular dermal matrix (Izol & Üner, 2019). A FGG is used as a one‐step or a two‐step technique for root coverage (RC; Deo et al., 2019), as it is used to increase the amount of attached gingiva (Izol & Üner, 2019). Platelet‐rich fibrin (PRF) is a second‐generation platelet concentrate that was introduced by Choukroun et al. in 2001 after platelet‐rich plasma (PRP) (Choukroun et al., 2006). It was developed as the first source of autogenous blood‐derived growth factors harvested without the use of anticoagulants (Miron et al., 2017). It contains various growth factors that are believed to contribute to periodontal regeneration including the platelet‐derived growth factor (PDGF), insulin‐like growth factor (IGF), transforming growth factor (TGF), epidermal growth factor (EGF), fibroblast growth factor (FGF), and bone morphogenetic protein (Bhushan et al., 2016). Injectable PRF (i‐PRF) is a liquid formulation of PRF (Turer et al., 2020). It has been produced by changing the centrifugation time, speed (700 r.p.m. for 3 min) and type of tube (Miron & Choukroun, 2017). The low‐speed concept may directly influence tissue regeneration by increasing fibroblasts migration, proliferation, and collagen messenger RNA levels (Fujioka‐Kobayashi et al., 2017). Injectable platelet‐rich fibrin may contribute to wound‐healing processes, as it is rich in platelets, leukocytes, and growth factors (Choukroun & Ghanaati, 2018). i‐PRF has many advantages, as it increases the total growth factors release, osteoblasts migration, and collagen‐1 synthesis (Ozsagir et al., 2020). It can stimulate tissue regeneration and it is used in regenerative treatments, with good outcomes (Izol & Üner, 2019). Fibronectin is an important component of i‐PRF with a high‐molecular‐weight extracellular matrix glycoprotein (Wang et al., 2019). It has a chemoattractant effect on fibroblasts and mesenchymal cells, and it is involved in many cellular processes, including tissue repair, and cell migration/adhesion that makes it used in periodontal regeneration as a root surface bio modifier (Bhushan et al., 2016; Izol & Üner, 2019). Biomodification procedures include using different agents to detoxify, decontaminate, and demineralize the root surface, thereby removing the smear layer and exposing the collagenous matrix of dentin and cementum, for example, citric acid, tetracycline HCl, EDTA, phosphoric acid, and fibronectin. In addition to laser systems, CO_2_ laser, PRP, and hyaluronic acid (Bhushan et al., 2016). Although a lot of studies reported applying various biomodification agents for root surface coverage, there is a lack of current randomized clinical trials—especially split‐mouth study design—comparing the effects of root biomodification with i‐PRF on RC, so this study was the first study that aimed to evaluate the efficiency of liquid PRF with FGG in Miller Class I or II GRs compared with FGG alone in split‐mouth study design.

## 2 MATERIALS AND METHODS

### 2.1 Study design, settings, and ethical approval

This randomized split‐mouth double‐blinded clinical trial has utilized a superiority design with a 1:1 allocation ratio to compare two techniques, consisting of FGG, with and without i‐PRF application in the treatment of deep (≥3 mm) recessions affecting the anterior teeth. This study was undertaken from March 2021 and December 2021 at the Department of Periodontology, Faculty of Dentistry, Damascus University. The study protocol, questionnaires, and informed consent are in full accordance with the ethical guidelines of the Declaration of Helsinki. The research project was ethically approved by the Local Research Ethics Committee of the Faculty of Dentistry (UDDS‐24082020/SRC‐2794). The project was self‐funded and it was registered at the ISRCTN registry under ID number: ISRCTN46963726. This randomized clinical trial has been written according to the new CONSORT statement.

### 2.2 Recruitment and eligibility criteria

Sixty patients aged between 19 and 40 years were referred to the Department of Periodontology during the study period because of the presence of GR in the upper and lower anterior teeth. The patients were investigated by the principal researcher (WA). The principal investigator searched for healthy patients who have bilateral deep GR with Class I or II according to Miller classification (≥3 mm in depth) at the buccal gingiva of upper or lower anterior teeth. A preoperative clinical examination was done to assess the probing depth and GR classification to determine the included cases. Those who met this condition were 48 patients. Thirty‐two patients were excluded due to the presence of systemic diseases that compromised their general immune status. Moreover, smoker patients, and those who were contraindications for surgery, or their recession defects associated with caries, deep abrasion restoration, or pulpal pathology were also excluded.

Therefore, 16 patients (10 females and 6 males) with 2 recessions on the contralateral anterior teeth of the same arch were included in the current research. All included patients, who accepted to participate in this study, signed an informed consent sheet after explaining all the details about the trial and the therapeutic part of it.

### 2.3 Sample size calculation

The study was powered to detect a minimum clinically significant difference of 1 mm in recession depth (RD) levels using *α* = 0.05, a power = 80%, obtained from previous studies (McGuire & Scheyer, 2010; Rajeswari et al., 2021). By using G Power 3.1 (Heinrich‐Heine‐Universität), as a minimum, 14 patients (28 surgical sites) were needed. The size was raised to 16 patients (32 surgical sites) to avoid any withdrawals.

### 2.4 Randomization

Randomization was performed by allocating each of the two recessions to one of the two techniques A (right side: FGG, left side: liquid PRF with FGG) or B (right side: liquid PRF with FGG, left side: FGG). The sequence of allocation was performed using a computer random generator (allocation ratio of 1:1). The sequence of allocation was concealed in opaque sealed envelopes, which were identified by the initials of the patient's name. Each patient's envelope was opened immediately before surgery.

### 2.5 Blinding

The presented study was double‐blinded; as the current study was an interventional study, the treating clinician could not be blinded regarding the technique used during procedures. However, the involved patients were completely blinded. The outcome assessor was also unaware of the patient's allocation during data analysis.

### 2.6 Clinical procedures

#### 2.6.1 Presurgical phase

**Figure 1:**
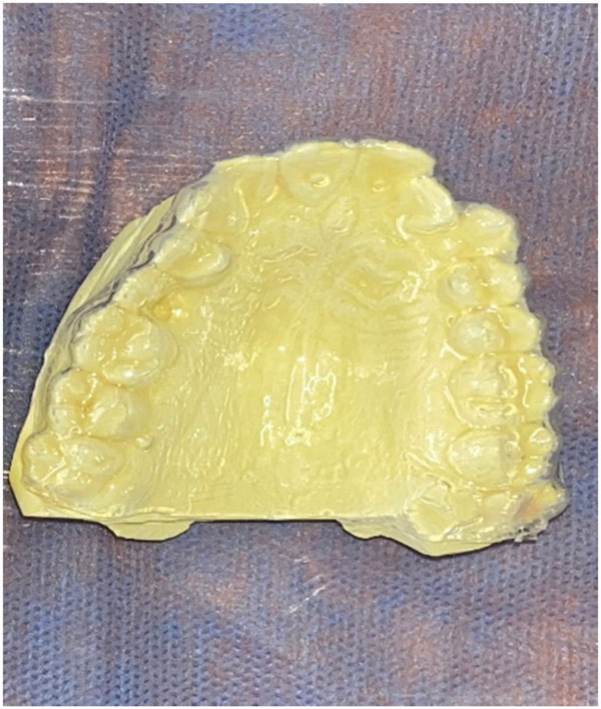
Vacuum plate.

**Figure 2:**
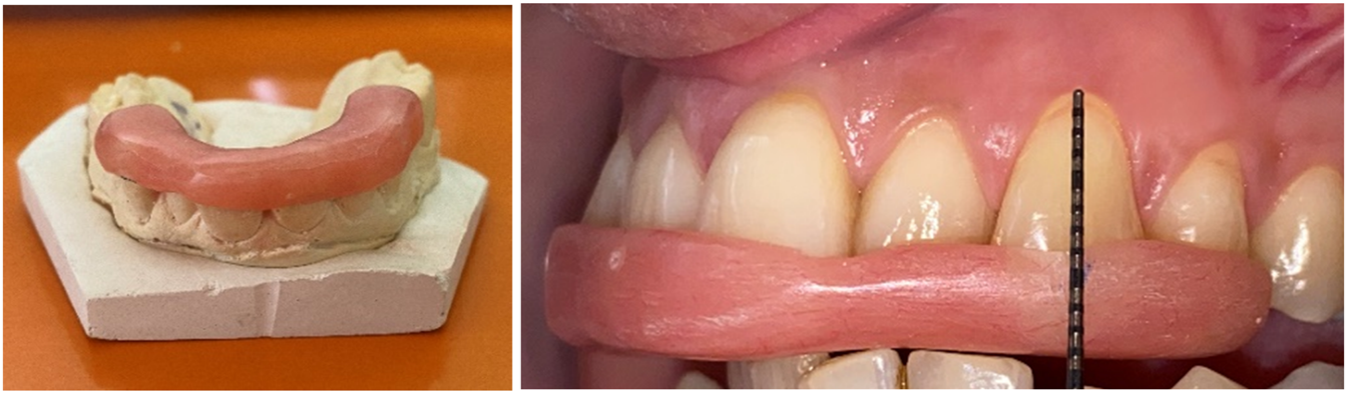
The acrylic stent.

After the selection of the patients, all of them received detailed instructions for oral hygiene. Scaling and root debridement were performed. Surgical treatment of the recession treatment defects was not documented until the participant could demonstrate a high standard of plaque control. The plaque index and gingival index were recorded (Silness & Löe, 1964) in a specially designed examination card for each patient. Alginate impressions were taken for the upper jaw to make a vacuum plate to protect the graft site after surgery (Figure 1). An acrylic stent was made for each patient to record the clinical measurements during the follow‐up periods to ensure the accuracy of recording measurements as described by Kumar et al. (2017) (Figure 2).

#### 2.6.2 Surgical phase

**Figure 3:**
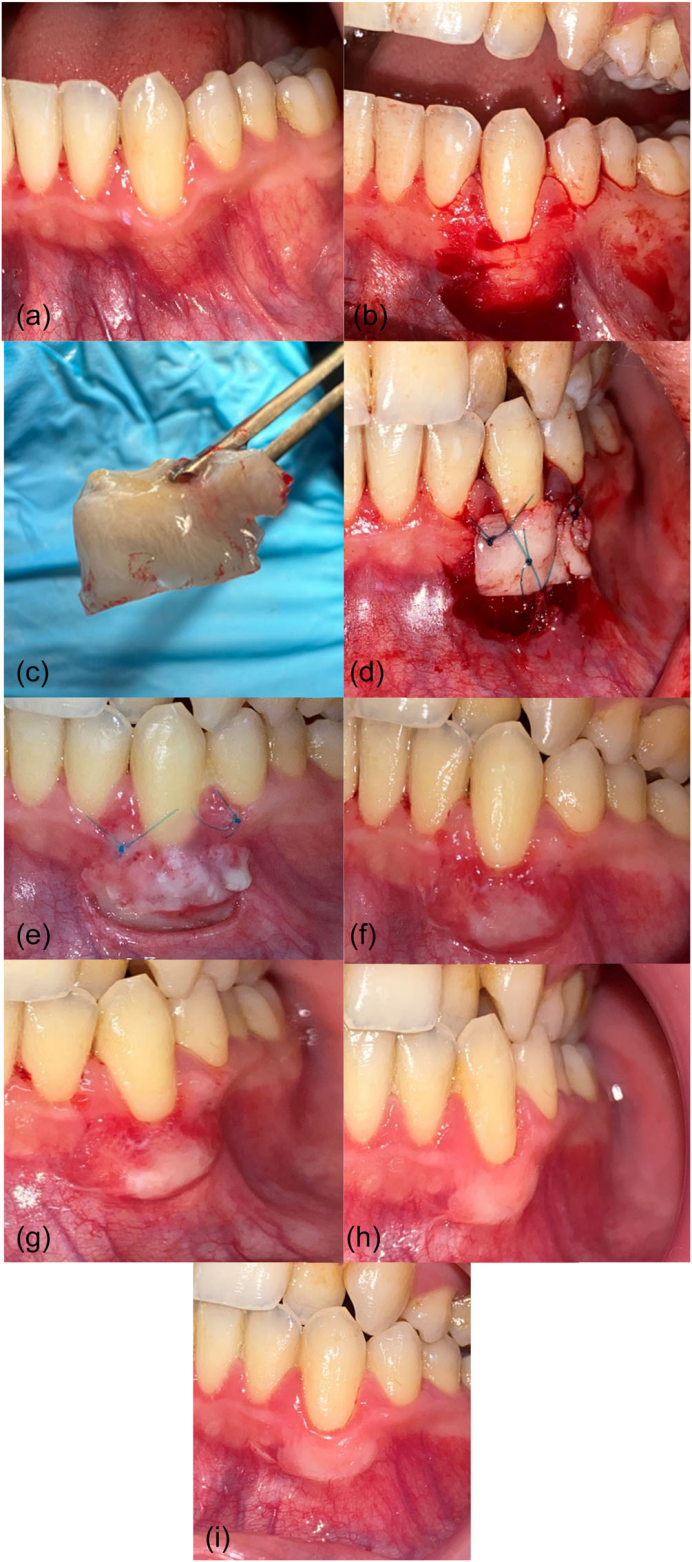
Control group. (a) Gingival recession (GR) on left canine. (b) Recipient site preparation. (c) Free gingival graft. (d) Suturing the graft in its place. (e) Healing after 1 week. (f) Healing after 2 weeks. (g) Healing after 1 month. (h) Healing after 3 months. (i) Healing after 6 months.

**Figure 4:**
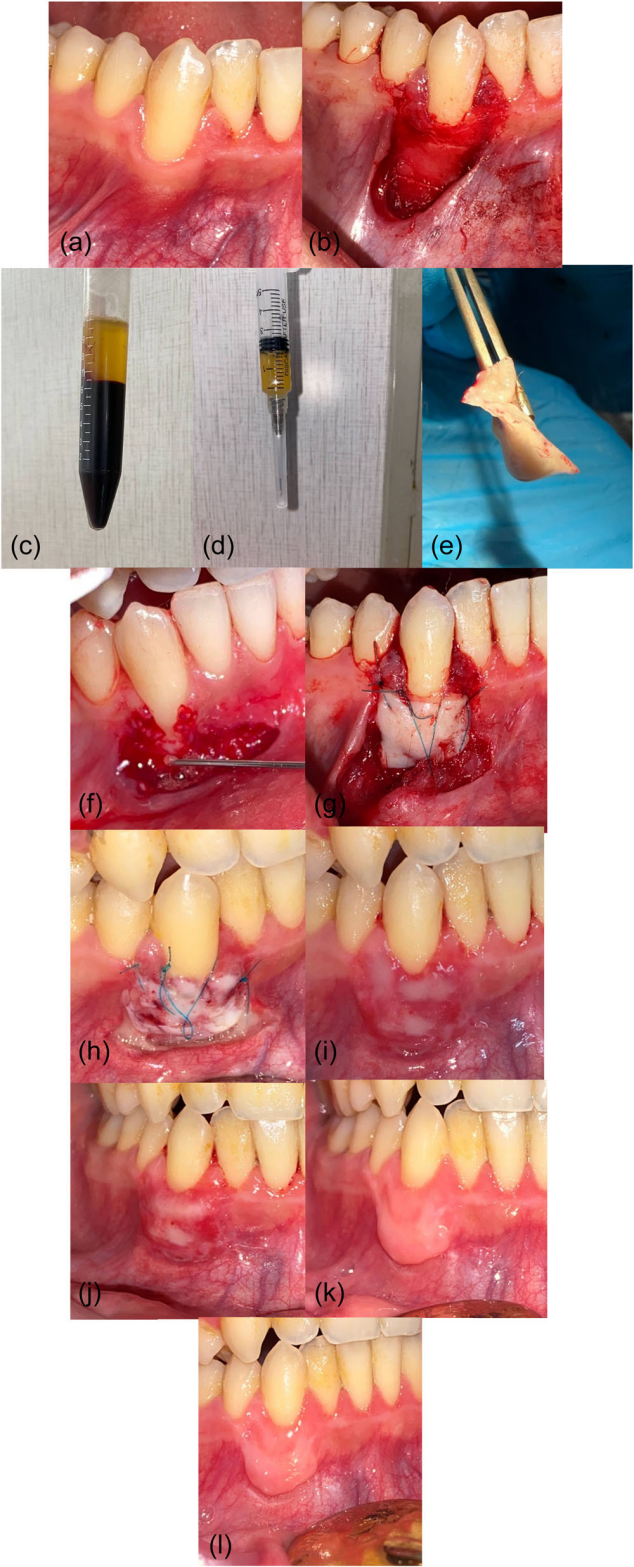
Test group. (a) Gingival recession (RR) on right canine. (b) Recipient site preparation. (c, d) Injectable platelet‐rich fibrin. (e) Free gingival graft. (f) Injectable PRF (i‐PRF) application on root surface. (g) Suturing the graft. (h) Healing after 1 week. (i) Healing after 2 weeks. (j) Healing after 1 month. (k) Healing after 3 months. (l) Healing after 6 months.

The surgical stage for each side was accomplished separately (right–left) at two different times, so that the surgical operation for the second side was done after ensuring the recovery of the first side, with a time difference of at least 1 month. The method of surgery described by Sullivan and Atkins (1968) was adopted. Sterilization and disinfection techniques were used for each patient. For the control group, the donor and recipient surgical sites were locally anesthetized with infiltration, using 2% lidocaine, 1:80,000 (Huons Lidocaine HCL). At the recipient site, horizontal incisions were made at the base of the interdental papillae adjacent to the site of recession and then two vertical incisions were made at the lateral ends of the horizontal incisions, extending beyond the mucogingival junction. A partial thickness flap was elevated and was deepened to a sufficient depth to stabilize the FGG in its bed. The dissected tissue was excised from the attached mucosa. Connective tissue was left over the existing bone to enhance blood supply and then the exposed root surfaces were smoothed with periodontal curettes (Hu Friedy®). The recipient site was washed with sterile saline and wet gauze was placed over the area until the graft was set in its place. After the injection of local anesthesia, the FGG was harvested from the palate from the area located lateral to the canine and mesial of the upper first molar. The thickness of the graft was ~1.5–2 mm. Then the vacuum plate was placed on the upper jaw with wet gauze inside it opposite the donor site to protect it. The graft was adapted to its recipient site and it was sutured to the intact keratinized gingiva with nonabsorbent nylon suture 4–0 (Pudong Jinhuan Medical Products Co., Ltd). Later, the pressure was applied gently with gauze soaked in saline for 2 min; no periodontal dressing was used at the recipient site. For the experimental group, the same steps were done, but before the stage of harvesting the graft, liquid PRF was prepared, 20 c.c.s. of the patient's blood were drawn, then it was collected in sterile conical bottom plastic tubes (15 mL) (Qingdao Carong Imp& Ex) without any additives, then the tubes were centrifuged using a protocol of 700 r.p.m. for 3 min (RCF‐max = 47 g) in a centrifuge (33° rotor angulation and 86 mm at the maximum Laboratory centrifuge EBA 200 series, Andreas‐Hettich‐gmbh& Co. KG) (Miron et al., 2018, 2019a) and when the centrifuge was over, the tube had contained orange i‐PRF at the top of the tube. Then, it was applied to the root surface and recipient site for 5 min before placing the FGG in its place (Figures 3 and 4).

#### 2.6.3 Postsurgical instructions

Amoxicillin was prescribed (500 mg, cap, 4 times per day for 7 days) and Brufen 600 mg was also prescribed at the beginning of the surgical procedure and subsequently when necessary. Patients were recommended not to brush their teeth in the treatment area but to wipe it with wet cotton with a chlorhexidine solution of 0.12% for a period of 2 weeks. After that, plaque control was performed by atraumatic tooth brushing. The sutures were removed after 10 days.

### 2.7 Outcomes measures

The patient was recalled to clinical examination at the end of the first week, after 2 weeks, after a month, 3 months, and 6 months.

#### 2.7.1 GR depth index (RD)

It was measured as the distance between the CEJ and the gingival margin.

#### 2.7.2 Width of attached gingiva index (AG)

It was measured by subtracting the sulcus depth from the distance between the free gingival margin and the mucogingival junction. Both RD and AG were performed at the mid‐buccal gingival of the teeth, by UNC‐15 probe (University of North Carolina‐15) with acrylic stent and recorded before starting, after 1 month, 3 months, and 6 months.

#### 2.7.3 Percentage of RC

It was calculated after 6 months according to the following formula (Shieh et al., 1997):

$$\mathrm{RC}=\frac{\text{Preoperative recession depth}–\text{Postoperative recession depth}}{\text{Preoperative recession depth}}\;x\;100$$

#### 2.7.4 Healing Index (HI)

It was recorded after 1 week, 2 weeks, and 1 month. HI by Landry, Turnbull, and Howley was used to evaluate the quality of the healing process (Gangwani et al., 2018; Pippi, 2017). Healing was recorded with a 5‐level score index ranging from 1 (*very poor*) to 5 (*excellent*) by a combination of the presence/absence of five clinical parameters (tissue color, response to palpation, granulation tissue, incision margin, and suppuration).

**Figure 5:**
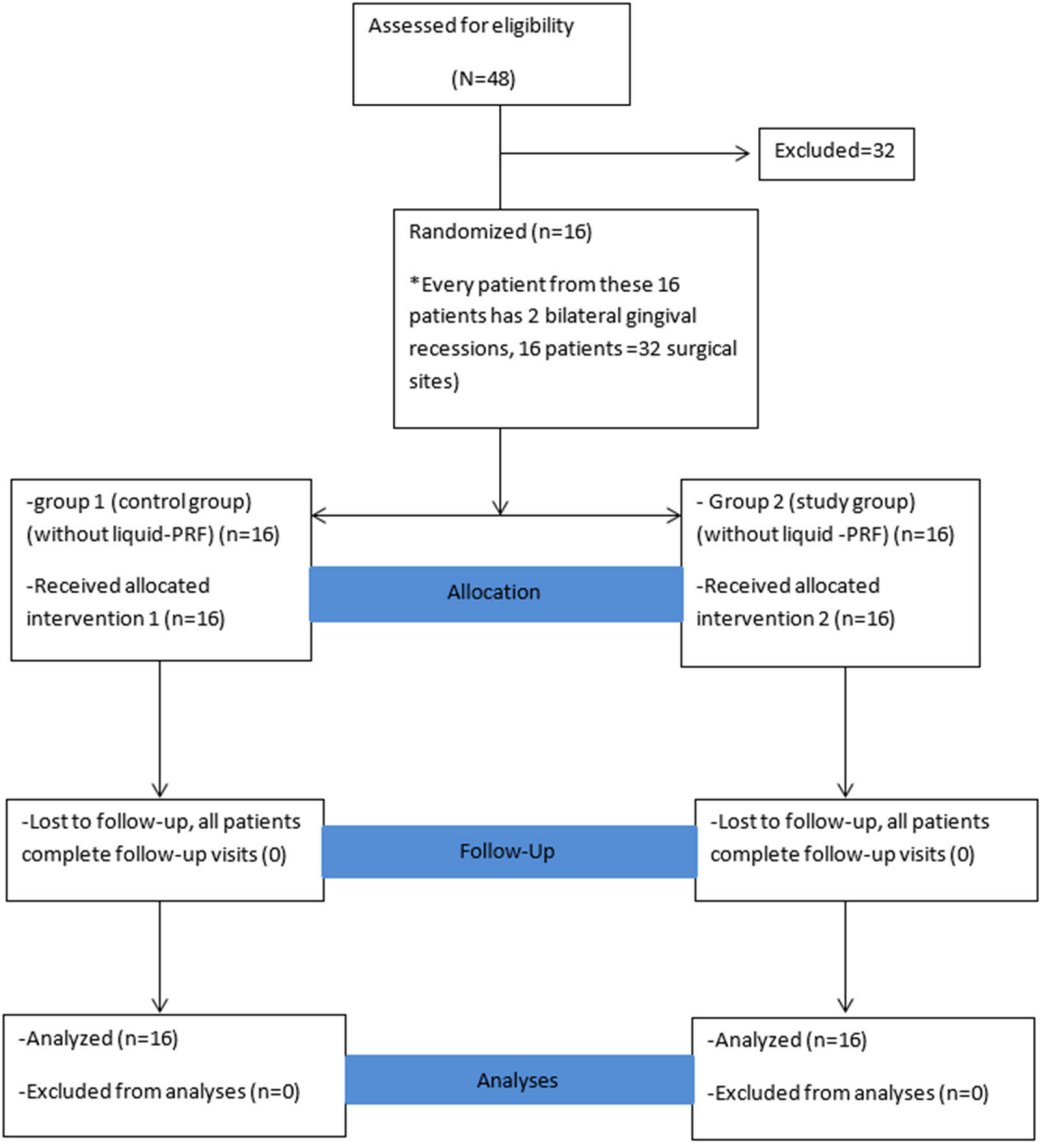
Flow chart of the patient.

The CONSORT flowchart diagram of the patients is reported in Figure 5.

### 2.8 Statistical analysis

Data were collected and recorded in Excel from Microsoft, then the statistical tests were done by using SPSS software (SPSS Version 22, IBM SPSS Inc.) with a significance level of 0.05. Mean and SD were used during the descriptive phase. Shapiro–Wilk test was used to evaluate if the quantitative measurements showed normal distribution. Mann–Whitney *U* test was used for intergroup comparisons during the study periods for RD, AG, and RC. *χ* ^2^ test was used for intergroup comparisons during the study periods for (HI).

## 3 RESULTS

A total of 16 bilateral GR patients (6 males and 10 females) were included in the study. The mean age of the patients was 35.6 ± 5.7 years.

### 3.1 GR depth index (RD)

Mean and SD values for RD in the study sample according to the treatment method and the studied period are shown in Table 1.

**Table 1:** Comparison of GR depth values between both groups.

Period	Groups	*N*	Mean ± SD	Mann–Whitney *U*	*Z* value	*p* a
Before treatment	Control group	16	2.88 ± 0.62	116.0	−0.557	0.578
	Liquid PRF group	16	2.75 ± 0.45			
After 1 month	Control group	16	1.00 ± 0.52	107.0	−0.942	0.346
	Liquid PRF group	16	0.81 ± 0.66			
After 3 months	Control group	16	1.00 ± 0.52	107.0	−0.942	0.346
	Liquid PRF group	16	0.81 ± 0.66			
After 6 months	Control group	16	1.00 ± 0.52	114.0	−0.646	0.518
	Liquid PRF group	16	0.88 ± 0.62			

Abbreviations: GR, gingival recession; PRF, platelet‐rich fibrin.

^a^ Mann–Whitney *U* test.

Mann–Whitney *U* test showed no significant differences between both groups during the studied time periods (*p* < 0.05 in each period). Before treatment, measurements were 2.88 ± 0.62 for the control group and 2.75 ± 0.45 for the experimental group (*p* = 0.578). After 1 month, measurements were 1.00 ± 0.52 for the control group and 0.81 ± 0.66 for the experimental group (*p* = 0.346). After 3 months, measurements were 1.00 ± 0.52 for the control group and 0.81 ± 0.66 for the experimental group (*p* = 0.346). After 6 months, measurements were 1.00 ± 0.52 for the control group and 0.88 ± 0.62 for the experimental group (*p* = 0.518) (Table 1).

### 3.2 Width of attached gingiva index (WAG)

Mean and SD values for WAG in the study sample according to the treatment method and the studied time period are shown in Table 2.

**Table 2:** Comparison of width of attached gingiva values between both groups.

Period	Group	*N*	Mean ± SD	Mann–Whitney *U*	*Z* value	*p* a
Before treatment	Control group	16	0.00 ± 0.000	128.000	0.000	1.000
	Liquid PRF group	16	0.00 ± 0.000			
After 1 month	Control group	16	3.75 ± 0.856	100.000	−1.142	0.253
	Liquid PRF group	16	4.13 ± 0.806			
After 3 months	Control group	16	3.19 ± 1.109	90.000	−1.533	0.125
	Liquid PRF group	16	3.88 ± 0.806			
After 6 months	Control group	16	2.75 ± 0.856	48.000	−3.256	0.001^b^
	Liquid PRF group	16	3.88 ± 0.806			

Abbreviation: PRF, platelet‐rich fibrin.

^a^ Mann–Whitney *U* test.

^b^ Significant difference.

Mann–Whitney *U* test showed that the WAG measurements before treatment were 0.00 ± 0.00 for both groups (*p* = 1.000). After 1 month, measurements were 3.75 ± 0.856 for the control group and 4.13 ± 0.806 for the experimental group (*p* = 0.253). After 3 months, measurements were 3.19 ± 1.109 for the control group and 3.88 ± 0.806 for the experimental group (*p* = 0.125). On the other hand, the WAG of the i‐PRF group was significantly better than the control group after 6 months (3.88 ± 0.806 and 2.75 ± 0.856, respectively [*p* = 0.001]; Table 2).

### 3.3 Percentage of RC %

RC was better in the liquid PRF group than the control group (70.83% ± 20.64%, 65.62% ± 18.48%, respectively), but this difference was not statistically significant (*p* = 0.587) (Table 3)

**Table 3:** Comparison of RC between two groups at 6 months.

Group	*N*	RC % after 6 months mean ± SD	Mann–Whitney *U*	*Z* value	*p* a
Control group	16	65.62% ± 18.48%	115.000	−0.543	0.587
Liquid PRF group	16	70.83% ± 20.64%			

Abbreviations: PRF, platelet‐rich fibrin; RC, root coverage.

^a^ Mann–Whitney *U* test.

### 3.4 HI

HI values for samples according to the treatment method and the studied period are shown in Table 4.

**Table 4:** Comparison of HI between both groups.

Period	Group	Healing index					*χ* ^2^	*p* a
		Very poor	Poor	Good	Very good	Excellent		
7th day	Control group	2	12	2	0	0	18.286	0.000^b^
	Liquid PRF group	0	2	12	2	0		
14th day	Control group	0	4	8	4	0	13.600	0.004^b^
	Liquid PRF group	0	0	2	12	2		
1 month	Control group	0	0	0	4	12	0.821	0.365
	Liquid PRF group	0	0	0	2	14		

Abbreviations: HI, Healing Index; PRF, platelet‐rich fibrin.

^a^ *χ* ^2^ test.

^b^ Significant difference.

The postoperative 7th and 14th days HI scores of the liquid PRF group were significantly better than the control group (*p* = 0.000 and *p* = 0.004, respectively), whereas there was no significant difference in HI scores between both groups in 1st month (*p* = 0.365) (Table 4).

## 4 DISCUSSION

GR has many problems, including tooth hypersensitivity, root caries, the unaesthetic appearance of the gums, periodontal attachment loss, and tooth loss (Jenabian et al., 2016). Several solutions have been offered to overcome the problem of GR, such as pedicle graft, connective tissue graft (CTG), coronally positioned flap, and FGG (Izol & Üner, 2019). Although most of these techniques result in significant clinical improvement, treatment of GR is still a challenge and researchers are working to find an effective method for this purpose (Jenabian et al., 2016). Although the majority of the published articles on Miller Class I and Class II recessions have demonstrated good results with SCTG but on the contrary, FGG was the best choice of the clinicians for the management this types of recessions especially in zones with inadequate width of attached gingiva and depth of vestibular fornix (Deo et al., 2019). In trying to improve RC, many studies reported applying various biomodification agents for root surface coverage, like citric acid, tetracycline HCl, EDTA, fibronectin, enamel matrix proteins, hyaluronic acid, and lasers (Bhushan et al., 2016; Deo et al., 2019). Biomodification determines the alterations of the disease root surface that would create an appropriate and hospitable surface for cell attachment, remove the smear layer and improve biocompatibility (Bhushan et al., 2016). Despite these benefits, the effects of biomodification agents on root surface coverage are still unclear (Oliveira & Muncinelli, 2012).

According to the search of the PubMed database, this is the first study that evaluates the effects of liquid PRF on wound‐healing, the width of attached gingiva, and RC with FGG in a split‐mouth study design. We used “ Liquid PRF“ term instead of” i‐PRF” in the current study based on the recommendations by Miron et al. (2019a). PRF is the concentration of platelets as constituents on the blood sample to support healing through several mechanisms, including angiogenesis, cell proliferation, and matrix remodeling by growth factors such as PDGF, TGF‐β, IGF, EGF, FGF, and bone morphogenic protein (Alpan & Cin, 2020; Karayürek et al., 2019; Kızıltoprak & Uslu, 2020). i‐PRF contains a higher number of regenerative cells and a higher concentration of growth factors because of the slower and shorter centrifugation spin (Ghanaati et al., 2014). Fibronectin is one of the components of i‐PRF (Grzesik & Narayanan, 2002) and it is an adhesive glycoprotein (Sevilla et al., 2010). FGG has some disadvantages like compromised blood supply, poor hemostasis, and problems in retention of the graft (Cohen, 2007), so the immobilization of the graft is important for the success of graft (Hassani et al., 2010). Thus, because of the features of liquid PRF, it was decided to use it as a root surface modifier (RSB) for the root surface. Similar to Izol's study (Izol & Üner, 2019), we applied liquid PRF on the root surface for 5 min before placing the FGG and waiting to be transformed into an adhesive form. A lot of studies have been conducted to improve RC with root surface biomodification (Damante et al., 2019; Deo et al., 2019; Murugan et al., 2021). In the study of Damante et al. (2019),  they used citric acid/tetracycline gel (CAT) and antimicrobial photodynamic therapy for RSB with CTG treatment, where they observed that both the CAT group and PDT group have higher RC (82.1% and 81.6%, respectively) in comparison with scaling and root planning group (57.7%) (Karayürek et al., 2019). Furthermore, Murugan et al. (2021) used Er, Cr: YSGG Laser for RSB with CTG to treat wide and deep GR defects, and they found better recession coverage despite a wide and deeper defect. Likewise, Dilsiz et al. (2010) used the Nd:Yag laser with CTG and they found that the RC was 33% in the test group and 77% in the control group. Moreover, Turer et al. (2020) used i‐PRF with CTG for the treatment of deep GR defects and they observed complete RC at 88% of the sites treated with CAF + CTG + i‐PRF and 80% of the sites treated with CAF + CTG. The current study had a similar result to Damante et al. (2019), Murugan et al. (2021), and Turer et al. (2020), where a significant reduction was observed in RD. Although recession decreased in both groups, better coverage was observed in the FGG + i‐PRF group. A FGG is widely used for increasing keratinized tissue dimensions to create an adequate zone of attached gingiva (Nabers, 1966). Moreover, it was observed an increase in the WAG in both groups after 1, 3, and 6 months. The difference between both groups was not statically significant after 1 and 3 months, but it was significant after 6 months. It may be explained by improved human gingival fibroblast cell migration, proliferation, and spreading by i‐PRF (Wang et al., 2017). Turer et al. (2020) observed a significant increase in WAG measurement when they use i‐PRF with CTG and CAF. Moreover, Kothiwale and Ajbani (2020) used chorion membrane along with PRF membrane to increase the WAG and they found that PRF enhances the healing during the follow‐up periods. Our current study corroborates this finding.

HI values of the liquid PRF group were significantly better than the control group on the 7th and 14th days. It may be explained by the high concentration of growth factors in PRF increasing the production of fibroblasts and myofibroblasts and inducing angiogenesis resulting accelerate early wound‐healing (Alpan & Cin, 2020). Moreover, TGF‐β, which is found in i‐PRF, takes important roles in various stages of palatal epithelium and connective tissue development and growth (Miron et al., 2017; Nawshad et al., 2004). Alpan & Cin (2020) evaluated the application of PRF to palatal tissue after CTG harvesting, and they found that PRF provided better wound‐healing clinically during 14‐day when compared with the control group (CTG donor site without PRF). It is important to pay attention to select an appropriate centrifugation tubes for the production of PRF as the tubes may affect to the final form of PRF; therefore, clot formation, cell behavior, and in vivo inflammation like tubes that containing silica and silicon, so we used plain nonchemical plastic tubes (Miron et al., 2021). Titanium PRF, which is prepared by using a protocol 2800 r.p.m. for 12 min, has histoconductive efficacy like CTG and it accelerates wound‐healing, so it may be good choice to treat this type of GRs, but it needs special equipments like titanium tubes so this was the limitation of use T‐PRF in this cases (Ustaoğlu et al., 2016). l‐PRF, which is prepared by using a protocol 2700 r.p.m. for 12 min, has the majority of platelets and leukocytes within the buffy coat with relatively no cells found within the first 4 ml of l‐PRF, whereas i‐PRF has the highest concentration of leukocytes/platelets (Miron et al., 2019a), so we used i‐PRF in our study. There are many improvements in centrifugation protocols like concentrated PRF (C‐PRF) and horizontal centrifugation (Miron et al., 2019a, 2019b). C‐PRF is a novel technique to isolate liquid PRF directly from buffy coat layer above the red cell layer by using l‐PRF protocols, it has 10‐fold increase in platelet and leukocyte yields (Miron et al., 2019b). The horizontal centrifugation in both solid (700 g × 8 min) and liquid (200 g × 8 min) formulations have higher concentrations and numbers of platelets and leukocytes when compared with fixed‐angle centrifuges, it needs a special centrifugation device (Miron et al., 2019a) and this is limitation to use this type of PRF in our country. This new preparations of PRF may have a positive effect on the results of GR treatments. Further studies with this types of PRF are required. A short follow‐up period time should be considered as a limitation, as 6 months may not be long enough to observe clinical results stabilization. Subjective aesthetic assessment is suggested to be important in GR treatment, so this is another limitation of this study. Another limitation is related to the small sample size because of strict conditions of inclusion criteria, so more reliable results could be achieved by considering these limitations. Moreover, the results of WAG measurement should be confirmed with further histological studies.

## 5 CONCLUSIONS

Within the above‐mentioned limitations of this study, it can be concluded that the addition of liquid PRF to the root surface with FGG showed further development in terms of decreasing RD, increasing WAG, and it accelerated wound‐healing. However, more studies with other platelet concentrate with FGGs are required to investigate the healing and width of attached gingiva.

## AUTHOR CONTRIBUTIONS

Wajeha Albatal conceptualized the idea, provided the treatment, and contributed to the writing and documenting. Tarek Qasem conceptualized the idea and supervised the master's thesis for Wajeha Albatal. Yasser Alsayed Tolibah contributed to the interpretation of data and the revision, formatting, and re‐editing of the manuscript. All authors read and agreed to the published version of the manuscript.

## CONFLICTS OF INTEREST STATEMENT

The authors declare no conflict of interest.

## ETHICS STATEMENT

The study was conducted according to the guidelines of the Declaration of Helsinki and approved by by the Local Research Ethics Committee of the Faculty of Dentistry (UDDS‐24082020/SRC‐2794). Informed consent was obtained from all subjects/caregivers involved in the study.

## Data Availability

De‐identified data are available upon written request to the corresponding author.
